# Noble Metals Functionalized on Graphene Oxide Obtained by Different Methods—New Catalytic Materials

**DOI:** 10.3390/nano13040783

**Published:** 2023-02-20

**Authors:** Mihaela Iordache, Anisoara Oubraham, Ioan-Sorin Sorlei, Florin Alexandru Lungu, Catalin Capris, Tudor Popescu, Adriana Marinoiu

**Affiliations:** 1 National Research and Development Institute for Cryogenics and Isotopic Technologies—ICSI, 4 Uzinei Street, 240050 Râmnicu Vâlcea, Romania; 2 Faculty of Chemical Engineering and Biotechnologies, 011061 Bucharest, Romania

**Keywords:** reduced graphene oxide (rGO), noble metal nanomaterials (NPs), catalytic activity

## Abstract

In recent years, research has focused on developing materials exhibiting outstanding mechanical, electrical, thermal, catalytic, magnetic and optical properties such as graphene/polymer, graphene/metal nanoparticles and graphene/ceramic nanocomposites. Two-dimensional sp^2^ hybridized graphene has become a material of choice in research due to the excellent properties it displays electrically, thermally, optically and mechanically. Noble nanomaterials also present special physical and chemical properties and, therefore, they provide model building blocks in modifying nanoscale structures for various applications, ranging from nanomedicine to catalysis and optics. The introduction of noble metal nanoparticles (NPs) (Au, Ag and Pd) into chemically derived graphene is important in opening new avenues for both materials in different fields where they can provide hybrid materials with exceptional performance due to the synergistical result of the specific properties of each of the materials. This review presents the different synthetic procedures for preparing Pt, Ag, Pd and Au NP/graphene oxide (GO) and reduced graphene oxide (rGO) composites.

## 1. Introduction

Polymer electrolyte membrane fuel cells (PEMFCs) represent one of the top researched power generation devices that convert the chemical energy of hydrogen directly into electricity [1]. Because this technology is clean as well as efficient, it provides an option for applications such as power generation on a large scale, co-generation of power and heat, backup and off-grid energy sources as well as transportation and mobile applications. Serving as one of the main problems that requires solving is the inferior kinetics of the oxygen reduction reaction (ORR) as well as the fuel cell reactions, which require a significant number of precious metals, leading to high costs of production. Poor performance and robustness of the catalysts being used as part of the electrodes is another significant problem to solve for PEMFCs [2]. Various materials and methods have also been proposed to solve these problems. Thus, one approach in this regard was replacing the carbon black in the catalyst layer with graphene. Over the last few years, numerous studies were carried out to evaluate the potential of using graphene-based materials in energy applications. Their large surface area, outstanding mechanical characteristics and superior electrical conductivity coupled with the workability of graphene have been the reasoning behind this attention [1]. Globally, there is a growing demand for nanostructured materials, as they are the key elements in enabling tools for renewable energy and hydrogen storage as well as indicator applications. Materials based on graphene have been shown to be very promising in achieving this goal due to their interesting properties, resulting in the conduction of a large number of theoretical and experimental studies [3]. With graphene being a flat monolayer of hexagonally arranged sp^2^-bonded carbon atoms bundled in a 2D figure-like network, it has been offered considerable attention from the experimental and the theoretical communities alike. Considering its superior electronic characteristics, graphene sheets offer potential solutions for the manufacturing of nanocomposites [4] and in the fabrication of field-effect transistors [5], dye-sensitized solar cells [6] as well as electrochemical sensors [7], electromechanical resonators [8] and lithium-ion batteries [4]. Graphene-based composite materials are capable of improving both electronic and thermal conductivity [9]. Moreover, graphene can provide stable mechanical support to active electrode materials, which can result in a potential composite for supercapacitor applications [10]. Noble metal nanoparticles (NPs) are a major topic for various fields such as electronics, sensing and catalysis as well as medicine and energy storage, as they exhibit unconventional physical and chemical properties [11]. In the last few decades, scientists have been especially interested in nano catalysis due to NPs satisfying nearly all the criteria of an ideal catalyst. They are of extremely small size and they present high surface-to-volume ratio, outstanding shape-dependent properties, uniformity in particle-size distribution while having a truly heterogenous nature and they are reusable with a near-constant efficiency [12]. The dispersion of metal nanoparticles on graphene sheets potentially offers a new way of developing catalytic, magnetic and optoelectronic materials [9]. However, the stability and catalytic activity of NP-based materials are limited due to their severe aggregation in aqueous media. More recently, graphene oxide (GO), which is a 2D material with a high specific surface area, when used as a platform to load NPs, has shown improvements in the stability and catalytic performance of the resulting NPs@GO nanocomposite [13]. Graphite oxide is a material that is produced through the oxidation of graphite leading to an increased interlayer spacing and the functionalization of its basal planes. Reproducible GO properties can be obtained depending on the state of preparation and on the graphene crystal, powders and particle size in relationship with the careful control of the water content of the applied acids, temperature conditions, length of time and drying conditions [3]. Graphene oxide (GO) is a two-dimensional carbon nanomaterial composed of carbon atoms organized in a hexagonal lattice, forming a flat sheet and containing oxygenated groups. GO has been a topic of interest for many applications (e.g., material science, nanomedicine, environmental technology and biotechnology) due to the extraordinary properties it exhibits, such as thermal resistance, electrical conductivity, optical transparency, chemical versatility and mechanical strength [14]. The GO-based materials also show remarkable properties, while chemical, thermal or other methods can be used in order to decrease the oxygen content, resulting in reduced graphene oxide (rGO). GO has been proven to be a very competent carbon support for the decoration of various metal nanoparticles due to it having significant surface area/active surface sites and its available nanosheets on both sides. The highly synergistic effect between nanoparticles and their uniform dispersion due to GO directly leads to an increase in the performance of the material [15]. The properties of both graphene and graphene oxide allows them to be used in several fields, especially for the preparation of nanocomposites and catalytic performances [16]. In noble metal/graphene nanocomposites using Pd, Pt, Au and Ag nanoparticles, the graphene oxide and metal salts are completely reduced. After decorating metal NPs on GO surfaces, the nonlinear optical properties can be significantly improved [3].

The novelty of this work is the comprehensive analysis of the state-of-the-art synthesis scalable methods for preparation of noble metals that are supported on graphene oxide. At the nano level, the compact atomic organization gives special properties for materials, such as electrical resistance or conductivity, precisely the properties for which noble metals are used. Noble metals represent limited group of metals including elements such as ruthenium, rhodium, palladium, silver, rhenium, osmium, iridium, platinum and gold. These elements are defined by two chemical properties that differentiate them from the rest of the metals—corrosion resistance and stability in oxidizing agents.

This work aims to update new synthesis methods of graphene functionalized with noble metals (Pt, Au, Ag and Pd) and to introduce new catalysts in fuel cell applications and renewable technologies. The paper undertakes an analysis of the recent research in the field and highlight the advantages of using noble metal nanostructures in catalytic applications. This new information comes in addition to works from the literature that focus on biomedical applications or biosensors [17], diagnosis or therapy [18] and less in fields such as fuel cell technologies, renewable energy and hydrogen storage [19,20]. Thus, Section 2 will address the theme “Methods of Synthesis NPs@GO Nanocomposites”, followed by Section 3 with examples of catalyst applications “Advantages and disadvantages of the synthesis methods of noble metals functionalized on graphene oxide” and ending with Section 4, “Conclusions”.

## 2. Methods of Synthesis NPs@GO Nanocomposites

**Figure 1:**
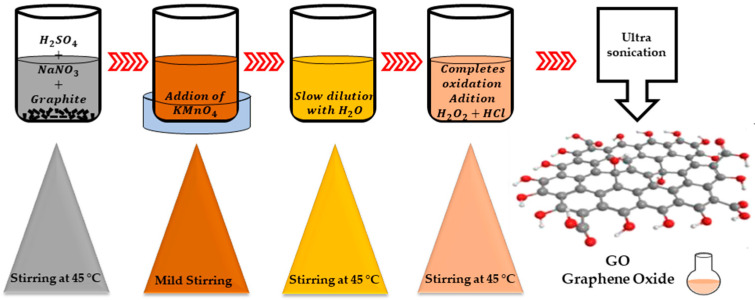
Hummer’s method.

The last allotropic form of carbon graphene has demonstrated that it possesses remarkable physicochemical properties (specific surface area between 1500–2600 m^2^ g^−1^) [21,22] that can be exploited for many electrochemical applications. Graphene possesses a single layer as a carbon sheet with the thickness of an atom. Graphene oxide (GO) represents the oxidized form of graphene and is considered in recent years as a suitable raw material for the manufacture of graphene. The most used method for obtaining graphene oxide, starting from graphite, is the Hummer chemical oxidation method. This method is shown schematically in Figure 1. This technique was also modified by other researchers in order to obtain an improved quality of graphene oxide. The obtained graphene oxide is subsequently dried and reduced using different reducing agents to improve electrical conductivity. To reduce graphene oxide, different reductants can be used (hydroiodic acid, hydrazine hydrate, hydrobromic acid, sodium borohydride, hydrochloric acid, sulfuric acid, ascorbic acid and dextrose) in order to eliminate the oxygen functional groups for the considerable improvement of electrical conductivity. The presence of different functional groups in graphene oxide influences the hydrophilic behavior, which is due to the level of oxidation. In addition, the multiple layers of GO are the consequence of a strong electrostatic charge but also of hydrophilicity, which can facilitate a good dispersion for anchored metals. The reduction in GO (specific surface area around 380–1000 m^2^ g^−1^, peak at 2θ = 11.2°) [21] is a classical method for obtaining a graphene-like structure. Reduced graphene oxide can be obtained by different chemical, thermal or photo-thermal reduction methods. Depending on the methods used, the produced rGO (specific surface area between 389.9–670 m^2^ g^−1^, peak at 2θ = 25.8°, electrical conductivity: 103.3 S·cm^−1^ and I_D_/I_G_ = 0.2) [23] approaches more or less close to the pure graphene structure. Among the reducing agents we mention inorganic chemical agents (for example, sodium borohydride) or organic (for example, phenyl hydrazine hydrate or hydroxylamine). Thermal reduction usually takes place in an inert atmosphere at elevated temperatures.

In order for graphene to be electrochemically active, it is often used to modify the carbon structure by functionalizing it with different types of noble metal elements. This functionalization will be dealt with in this review.

The reported methods aimed at the synthesizing of different metal nanoparticles (MNPs)/rGO nanocomposites involve chemical reduction, electrochemical deposition, thermally assisted method, photochemical reduction, microwave irradiation and sonochemical reduction. In most of these methods, a suspension of GO based on water or ethanol is allowed to react with a metal precursor in the presence of a reducing agent such as hydrazine hydrate, ascorbic acid, sodium citrate or glucose in order to obtain a simultaneous reduction in both the metal ions and the GO resulting in the production of MNPs/rGO composites.

### 2.1. Chemical Reduction

**Figure 2:**
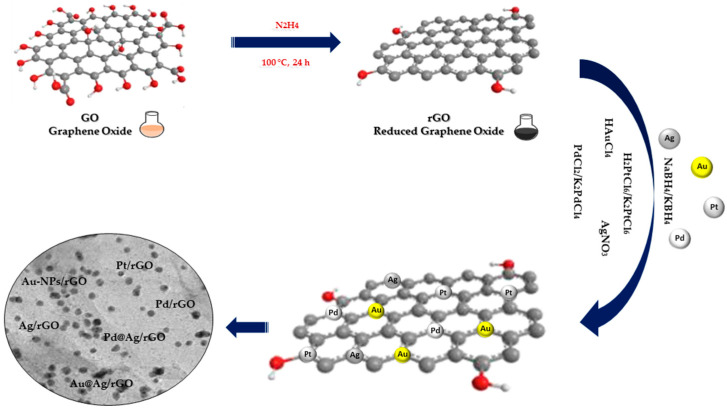
Chemical reduction synthesis of noble metal nanocomposites.

Chemical reduction is the most commonly used method to effectively immobilize NPs on GO and rGO. This method involves noble metal ions in solution being reduced to NPs on GO nanowires through additional reductants such as NaBH_4_, ascorbic acid, sodium citrate or hydrazine (Figure 2). Usually, the GO and rGO dispersion is firstly mixed with noble metal salt solutions, following which the noble metal ions begin adsorbing on the GO and rGO nanosheet surface through electrostatic interaction. Following this, the reducing agents in the mixture reduce the noble metal ions adsorbed in NPs on GO and rGO nanowires [24].

The three fundamental steps constituting the reduction process are as follows: (1) adsorption/reduction, (2) nucleation and (3) growth. The presence of oxygen-containing functional groups on the surface of the GO and rGO favors the adsorption of free metal ions through electrostatic interactions, followed by the reduction in metal ions by a reducing agent and finally the growth of NPs on the GO and rGO sheets. In spite of the formation of MNPs by chemical reduction being a facile process, this technique is limited due to difficulties sterned from size and morphology of the NPs, which can potentially result in polydisperse and large sizes of on GO and rGO surfaces [18].

Guo et al. [24], through the use of the chemical co-reduction method, synthesized a series of Pt-Ni/rGO catalysts with different Pt/Ni molar ratios and a total metal concentration of 0.99 mM. The production of these catalysts was performed through the rapid injection of KBH_4_ to reduce H_2_ PtCl_6_ and Ni(NO_3_)_2_ in the experiment at a temperature of 273 K and in an N_2_ atmosphere. X-ray powder diffraction (XRD) was used for the characterization of the detailed crystalline phases of the Pt-based NPs loaded on partially reduced GOs. The XRD results for the Pt/rGO catalyst displayed four diffraction peaks of (111), (200), (220) and (311). The peaks seen in the Pt_40_-Ni_60_/rGO catalyst model were shifted to a high angle range suggesting the formation of ally structured Pt-Ni BNPs loaded on rGO. Transmission electron microscope (TEM) images clearly visualized the uniform distribution of the Pt and Pt-Ni NPs on the rGO with an average size of about 3.4 and 2.6 nm; these results were consistent with the ones from the Scherrer formula. This indicates that the size of the Pt_40_-Ni_60_ BNPs of Pt_40_-Ni_60_/rGO catalyst could be reduced through the doping of Ni. Furthermore, the lattice stripe spacing of 0.220 nm can be assigned to the (111) lattice planes of Pt. The HRTEM image of Pt_40_-Ni_60_ BNPs showed a lattice stripe spacing of 0.206 nm, value which is intermediary between the lattice spacing of crystalline Pt (111) at 0.226 nm and Ni (111) at 0.203 nm. This serves as a further indicator that the rGO-supported Pt-Ni BNPs possess an alloy structure. Moreover, elemental mapping images of the catalyst revealed that the C and O elements were uniformly distributed in the entire sample, and that Pt and Ni elements were aggregated into particles, thus serving as confirmation of the formation of alloy-structured Pt-Ni BNPs. After an XPS characterization of the Pt/rGO catalyst, the C 1s spectrum typically showed four peaks at 284.75, 284.86, 286.9 and at 287.6 eV, which assigned the characteristics of C-C, C=C, C-O and C=O, respectively. The fact that O 1s XPS spectrum can be deconvoluted into peaks corresponding to O=C (at 531.7 eV) and O-C (at 532.88 eV) can be used as an explanation for the incomplete reduction in the function groups (hydroxyl, carboxyl, etc.) of GO during the co-reduction process. Pairing peaks assigned to metallic Pt^0^ 4f(_7/2_) and Pt^0^ 4f(_5/2_) were located at 71.32 and 72.2 eV in the Pt 4f spectrum with one other pairing peak assigned to oxidized Pt^(2+)^ 4f(_7/2_)and Pt^(2+)^ 4f_(5/2),_ being located at 72.2 and 75.62 eV, respectively. The first pairing peaks in the Pt/rGO catalyst were found to have higher binding energies than the bulk Pt 4f_(7/2)_ and Pt 4f_(5/2)_ (71.1 eV and 74.4 eV, respectively) by approximately 0.22 eV and 0.1 eV, which is an indicator that the Pt NPs were positively charged.

In another study [25], cobalt was used in the fabrication of AgPdNPs supported on rGO. Firstly, NaBH_4_ was used to reduce Pd ^(2+)^ and Co ^(2+)^ with the resulting amorphous Co_3_(BO_3_)_2_ and AgPd on the surface of the rGO. The amorphous Co_3_(BO_3_)_2_ can be removed simply through etching with H_3_PO_4_. Using this method, the prevention of the aggregation of AgPd nanoparticles is effective, thus obtaining well-dispersed AgPd nanoparticles. The rGO catalyst supported by the AgPd nanoparticles was used for the catalytic transfer hydrogenation of nitro-compounds at room temperature using H-COOH as hydrogen donor. This obtained catalyst has a high catalytic efficiency, which is a result of the highly dispersed bimetallic nanoparticles coupled with the synergistic interaction between the metallic nanoparticles and the support. TEM showed that the AgPd particles of Ag_0.1_Pd_0.9_/rGO catalyst were effectively dispersed on the rGO lamellar support. The obvious lattice fringe found in the representative high-resolution TEM image of Co_6_Ag_0.1_Pd_0.9_/rGO demonstrated the good crystallinity of the AgPdNPs. It resulted in a lattice spacing of 0.23 nm, value between the (111) lattice spacing of face-centered cubic Ag at 0.24 nm and Pd at 0.22 nm. This implies that AgPd has formed as an alloy structure. It was found that the form of the AgPd nanoparticles was not uniform; however, there was a narrow size distribution averaging at 4.3 nm ± 0.9 nm. The XRD patterns of AgPd hybrids in Co_6_Ag_0.1_Pd_0.9_/rGO catalysts observe a diffraction peak located between the Ag (111, 2 theta = 38.03 deg) and Pd (111, 2 theta = 40.10 deg) diffraction peaks, further suggesting the formation of the AgPd alloy. As AgPd has been incorporated by Co_3_(BO_3_)_2_, no AgPd peak of Co_6_Ag_0.1_Pd_0.9_/rGO could be observed, which is further attested by the TEM results. A nitrogen adsorption/desorption analysis was carried out at 77 K in order to determine the porosity of Ag_0.1_Pd_0.9_/rGO and Co_6_Ag_0.1_Pd_0.9_/rGO. Co_6_Ag_0.1_Pd_0.9_/rGO was found to have a low specific surface area of approximately 6 m^2^ g^−1^, which was attributed to the high amount of Co_3_(BO_3_)_2_ occupying the surface of the rGO. By comparison, in Co_6_Ag_0.1_Pd_0.9_/rGO, an increased absorption of nitrogen (310 m^2^ g^−1^) can be observed. After removing Co_3_(BO_3_)_2_ by etching with H_3_PO_4_, (Co_6_)Ag_0.1_Pd._0.9_/rGO results in high porosity, which serves in facilitating reactant diffusion to the metal nanoparticles. (Co_6_)Ag_0.1_Pd_0.9_/rGO presents a higher surface area (278 m^2^ g^−1^) than Ag_0.1_Pd_0.9_/rGO (135 m^2^ g^−1^). XPS measurement results showed that Ag and Pd alike in the (Co_6_)Ag_0.1_Pd._0.9_/rGO catalyst are found in reduced states. The electronic states of Pd^0^, 3d5/2 and 3d3/2 can be detected at 335.83 eV and 341.11 eV, respectively. The spectra of the Ag 3d and Pd 3d observed attest that (Co_6_)Ag_0.1_Pd_0.9_/rGO is made of metallic Ag and Pd, serving as additional confirmation of the effective synthesis of the AgPd alloy. Pd^2+^ peaks were detected at 338.14 eV and 343.48 eV, which can be attributed to the oxidation of metallic Pd in an environment containing oxygen.

Abbasi and co-workers [26] synthesized Pd nanoparticles using PdCl_2_ and NaBH_4_ as a strong reducing agent and polyvinyl alcohol (PVA) as stabilizing agent. During the chemical process, the palladium Pd^2+^ from the salt solution was reduced to Pd° as nanoparticles. A total of 120 mL of distilled water, 0.88 mL of freshly prepared 2% polyvinyl alcohol (PVA) solution and 2.15 mL of 0.02 M PdCl_2_ solution were immediately added, resulting in a yellow-brown solution. A 0.1 M NaBH_4_ solution (0.86 mL) was then slowly added to the reaction mixture while stirring vigorously. Observing an immediate color change to brown served as an indicator for the formation of Pd NPs. Following UV–Vis spectroscopic analysis, the palladium nanoparticles present the absorption spectrum of 4.1 p.m. It was found that the absorption peak of the Pd^2+^ precursor no longer appears at 420 nm, with this peak disappearing being an indicator for the complete reduction in the Pd^2+^ nanoparticles to Pd°. After three measurements, a size of 122 nm was found. The zeta potential of the palladium nanoparticles was immediately at −3.91 ± 3.85 mV, as shown. Regarding the stability of Pd NPs, although the zeta potential showed lower values, the synthesized nanoparticles were stable at room temperature and did not show any sign of agglomeration during the last 12 months.

Shu et al. [27] used a mildly tempered process of oxidation to obtain the in situ oxidation of PdIr alloy on NGs (nitrogen-doped graphene), (PdIrO/NGs). K_2_PdCI_4_ (36.6 mg) and H_2_IrCl_6_·6H_2_O (7.7 mg) were dispersed in deionized water and then mixed under strong stirring. The obtained solution was added to the NGs dispersion with stirring, and then the NaBH_4_ mixture was slowly dispersed into the aqueous solution. The obtained dispersion reacted in an ice bath under an N_2_ atmosphere for 300 min. The powder resulting from vacuum filtration, washing and lyophilization was calcined at 250 °C to obtain PdIrO/NGs. The same steps excluding calcination were used to synthesize the PdIrO/NGs catalyst. The XRD patterns of PdIrO/NGs, PdIr/NGs and Pd/NGs showed a broad peak between 20° and 30°, which was identifiable as the proprietary peak of NGs. The two diffraction peaks of 39.4° and 45.8° for Pd/NGs were found to correspond to the (111) and (200) planes of metallic Pd, respectively. The pattern revealed through XRD of PdIrO/NGs, which evidently showed a positive shift of the diffraction peak between 39.4° and 40.7° in comparison to the peak of Pd/NGS, it was an indicator of the high formation quality of the Pd-Ir alloy. In addition, in the XRD pattern of PdIrO/NG_S_, three diffraction peaks can be clearly observed at 33.9°, 41.9° and 54.8°, which correlate to the (101), (110) and (112) planes of PdO. Following the XPS analysis, a deconvolution of the Pd 3d spectra was discovered into two groups at 340.6 eV 335.3 eV, whereas the peaks discovered at 342.4 eV and 336.9 eV in the spectra may relate to PdO/_2/2_ and Pd 3d_5/2_. A significant increase in the concentration of PdO and IrO_2_ in PdIrO/NG_S_ can be observed, meaning that the alloy was oxidized. TEM analysis showed a uniform monodispersion of the nanoparticles on the NGS surface with a slight decrease in particle size observed after alloy formation and with a significant increase following calcination. EDS mapping demonstrated the homogeneous dispersion of C, N, O, Pd and Ir in PdIrO/NG_S_. BET (Brunauer–Emmett–Teller type IV) isotherms indicated that additional mesopores can be obtained in PdIrO/NG_S_, as they are in agreement with the pore dimension distribution curves. The largest specific surface area is that of PdIrO/NG_S_, with a higher limit of 122.4 m^2^ g^−1^ while PdIrO/NG_S,_ with a value of 96.9 m^2^ g^−1^ and with a value of 40.9 m^2^ g^−1^, have lower surface areas. This demonstrates that large BET surfaces and meso-porous structure are used to improve electrocatalytic activity.

Teffu et al. [28] synthesized Pd-rGO using electroless palladium deposition by immersing the rGO in a sodium hypophosphite-based plating bath. The electroless plating bath, which contained 50 mL of sodium hypophosphite (10 g L^−1^) as reducing agent and 5 g of rGO, was subjected to constant stirring (300 rpm) for 30 min at 50 °C, followed by adding PdCl_2_, 160 mL of NH_4_OH (28%) and 27 g of NH_4_Cl, respectively. A total of 50 mL of plating solution was added to the bath solution and the mixture was stirred for 30 min to allow Pd plating on the surface of the rGO sheets. The mixture was filtered, washed with ultrapure water and dried overnight at 80 °C. As observed from the XRD patterns in the case of Pd-rGO, the characteristic peak at 2θ = 24° is attributed to the (002) planes of the chemically reduced GO stack, which is an indicator for the effective reduction in GO by hydrazine hydrate. At the same time, it was found that the two diffraction peaks centered at 2θ of 39 and 45° can be attributed to the (111) and (200) reflections of the Pd nanoparticles, respectively. Investigation on the thermal stability of the fabricated Pd-rGO nanocomposite was performed using thermogravimetric analysis (TGA), with the results concluding that Pd-rGO shows a 15% loss of weight for the entire investigated temperature range (up to 560 °C). At 100 °C, the slight lost weight was caused by the loss of adsorbed water, while the losses of up to 400 °C are a result of the decomposition of residual hydroxyl and carboxyl functional groups. The FTIR spectra of Pd-rGO nanocomposite showed, as is expected, either the disappearance or the significantly reduced intensity of the FTIR peaks belonging to rGO after the reduction process, which serves as confirmation of the formation of Pd nanoparticles on rGO. Further observation showed very low band intensities in Pd-rGO, with some even disappearing, with reference to the rGO spectrum. This implied that the palladium incorporation on the rGO is on the surface of the graphene oxide sheets.

Rajkumar et al. synthesized [29] NP Au@Pt by two chemical methods. Au@Pt NPs were initially synthesized using Fren’s method: HAuCl_4_ was placed in a triple-neck flask connected to a condenser under strong stirring. This was heated to a boil, following which a solution of sodium citrate was quickly inserted and then the mixture was brought to a boiling temperature again. The boiling process was maintained for 10 min, with stirring being maintained for an additional 15 min post removal of the heat source. This solution was then naturally cooled to room temperature. Seed-mediated growth was the second method used to synthesize Au@Pt NPs. H_2_PtCl_6_, deionized water and prepared Au-NPs were mixed in a beaker. This obtained mixture was refrigerated afterward to 4 °C followed by slowly adding NaBH_4_ under stirring to obtain Au@Pt NPs. The nanostructures and morphology of multi-walled carbon nanotubes (MWCNTs) and GO/MWCNT dispersion were conducted by TEM and showed interlaced tubular structures with an average diameter of approx. 20 nm. Several lamellar structures integrated with carbon nanotubes (CNTs) could be observed upon the supplementation of GO, essentially scattering the CNTs. The Au-NPs showed a typical spherical structure with a particle diameter of approximately 13 nm. The reduction by hydroxyl groups of chloruretic ions on sodium citrate resulted in the formation of Au NPs. Synthesizing Au@Pt NPs resulted in a slightly increased particle size while for GO/MWCNT/Au@Pt NPs, the successive drop method was employed in the copper mesh. Au@Pt NPs were pre-applied on the GO/MWCNT surface, thus a new three-dimensional sensing interface for glucose sensing was constructed. Spectra obtained through Raman of MWCNTs, GO/MWCNTs and GO/MWCNTs compatible with Au@Pt NPs showed all characteristic Raman bands of MWCNTs at 1572 cm^−1^ and 1345 cm^−1^ relating to G and D bands. D comes from the disordered carbon structure, and the G band from sp^2^ hybridized carbon atoms. The intensity ratios (I_G_/I_D_) of the two peaks (G and D) have different values for different samples, with 0.93, 0.85 and 0.72 for MWCNT, GO/MCNT and GO/MWCNT/Au@Pts NPs, respectively. The escalation in ID indicated an expansion of the disordered carbon structure when supplementing GO and Au@Pts NPs, which demonstrated the effective construction of GO/MWCNT/Au@Pt NPs.

### 2.2. Thermally Assisted Method

**Figure 3:**
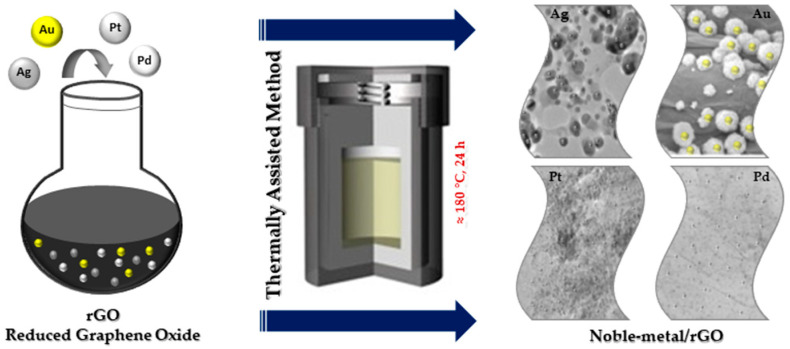
Thermally assisted synthesis of noble metal nanocomposites.

The thermally assisted method is one of the important methods used to fabricate NPs@GO nanocomposites more simply at high temperature (Figure 3) [13]. Thermally assisted synthesis is an easy and efficient method used to immobilize NPs on GO. The speed of the process makes the size and the distribution of the NPs@GO, in this case, difficult to control.

Abdulhusain et al. [30] prepared Ag-ZnO-rGO ternary nanocomposites by an in situ hydrothermal process in the presence of 1,8-diamino-3,6-dioxaoctane (DDO). The nano-photocatalysts possessing attractive physicochemical properties led to the idea of using a different procedure for the enhancement of Ag-ZnO-rGO nanocomposite properties in applications related to water treatment. Preparation of the appropriate nanocomposites at a lower temperature and for a shorter time have led to in situ synthesis being the chosen method. A 1,8-diamino-3,6-dioxaoctan was utilized in the synthesizing of the ternary Ag-ZnO-rGO nanocomposites, since the generous DDO carbon chain acts as a limiting factor to the accumulation of nanostructures. Firstly, graphene oxide was dispersed in distilled water resulting in solution A. Following this, zinc nitrate hexahydrate was in an aqueous mixture containing DDO, which resulted in solution B. The next step was the preparation of the silver nitrate aqueous solution. Finally, solutions A and B were mixed, while also pouring ethanol into the mixture. The resulting transparent suspension was placed in an autoclave and conditioned at 140 °C for an interval of 120 min. The collected precipitate was washed several times with distilled water and ethanol before being dried. The percentage weight of Ag was adjusted in order to attain the product with the highest performance. Moreover, studies were conducted on the effect of 1, 8-diamino-3, 6-dioxaoctan on the size distribution, morphology and purity of the product. Finally, the nanocomposite was exposed to degradation using a pollutant (RhB—rhodamine-B) in order to study its photocatalytic activity. Studies were performed to evaluate the changes in photocatalytic performance of the product by varying the pH and the concentration of Ag and the dye itself, respectively. The largest percentage of dye degradation was observed at a concentration of 10 ppm, with the pH of the mixture regulated to 11. The increased performance of the photocatalyst in alkaline media can be attributed to absorbed hydroxyl anions at the surface of the photocatalyst. Based on kinetic studies, photocatalytic reactions follow pseudo-first order with holes and hydroxyl radicals being critical active agents for photocatalysis. It is worth noting that the used synthesis process for this type of nanocomposite can be further applied for the rest of the nanocomposites due to its simplicity and eco-friendliness.

Rudra et al. used the thermally assisted method in synthesizing Au-Mn_3_O_4_-decorated graphene oxide and Au-Mn_3_O_4_ nanocomposite [31]. It was synthesized as follows: GO was added to distilled water in a beaker and sonicated for 30 min and this solution was then placed into screw cap tubes. Afterward, manganese acetate was added to the reaction mixture and the gold (III) solution. In order to balance the pH, sodium acetate was added to the solution. The screw cap tubes were stored in modified hydrothermal (MTH) conditions for 24 h. The sample was then washed several times with deionized water before being dried under vacuum to result in the Au-Mn_3_O_4_-decorated GO nanocomposites. The XRD results showed that the diffraction peaks correspond to (101), (112), (200), (103), (211), (204), (105), (303), (321), (224), (116), (305) and (413) planes of the Mn_3_O_4_, respectively. The peak positions at 2θ = 38.45°, 44.34°, 64.47°, 77.35° and 81.70° support the presence of Au (0) with the corresponding planes being 111, 200, 220, 311 and 222. Raman analysis shows a sharp peak at 658 chm^−1^ for the Au-Mn_3_O_4_/GO composites, which was attributed to the Mn_3_O_4_ decorated on the GO. The material’s morphological features and elemental contents were investigated using TEM and EDX analysis. The GO image shows the typical flake-like layered material whereas the image of the AuMn_3_O_4_ composite has a nanorod morphology owing to the growth of Mn_3_O_4_ nanorods from the Au (0) nucleation centers.

Wang et al. presented the preparation of Ag/CeO_2_ anchored on reduced graphene oxide (rGO) nanocomposite. Ag/CeO_2_-rGO is considered to be a simple, recyclable and sustainable photocatalyst for the esterification of aldehydes at room temperature under visible light irritation [15]. The catalyst was prepared as follows: 5 mmol of Ce(NO_3_)_3_·6H_2_O and 0.5 mmol of AgNO_3_ were slowly added to 1.8 mg mL^−1^ GO aqueous solution with constant stirring and followed by the addition of 0.5 mmol polyethylene glycol molecular weight 4000 (PEG 4000) and 20 mmol urea. The reaction mixture was transferred to an autoclave for the hydrothermal reaction at 185 °C for about 24 h. After cooling in the autoclave, the obtained solid sample was washed with deionized water and ethanol, then dehydrated at 50 °C in a hot air oven for 12 h. XRD analysis showed two additional peaks at 25.5 and 38.19 degrees, corresponding to the (002) plane of rGO and (111) planes with metallic phases of Ag. The specific surface area of reduced graphene oxide-embedded Ag/CeO_2_ was found to be 292.6 m^2^/g with a maximum of the pores in ranges from 5 to 15 nm. 

Das et al. synthesized using thermally assisted method Pt-M/GNPs (graphene nanoplatelets) (M = Ni, Fe and Cu) of catalysts. The adsorption isotherm showed that the use of 0.2 g of metal precursor over 0.1 g of GNPs yielded the highest metal loading [1]. Pt-M/GNPs catalysts were characterized from a physical point of view using XRD analysis, thermogravimetric analysis (TGA), inductively coupled plasma mass spectrometry analysis (ICP-MS), high resolution transmission electron microscopy analysis (HRTEM) and Raman analysis. XRD was used on the synthesized catalyst samples to determine the development of metal crystal structures on the GNPs support. The samples were scanned between 10° < 2θ < 90°. The distinct diffraction for the Pt/GNP catalyst corresponds to the (111), (200), (220) and (311) planes of Pt in the face-centered cubic crystal structure. TGA analyses were carried out between at a temperature ranging from 25 to 1000 °C with a 10 °C min^−1^ heating rate under air atmosphere. TGAs were used on the GNPs support material in order to determine the total metal loading. The results showed that the weight content of the metal nanoparticles (Pt-Ni, Pt-Fe and Pt-Cu) were approximately 24.0–30.0 wt%. ICP-MS analysis was employed in order to establish the composition of the Pt-M/GNPs catalysts. The results showed that the Pt loading (% by weight) was between 17.5% and 25.4% and the metal loading (% by weight)was 3.40% Ni, 1.40% Fe and 3.20% Cu. The particle size and morphology were investigated using TEM, considering its capability for imaging at an atomic scale. The average particle size was found to be 1.7 nm for Pt-Ni/GNPs, 1.6 nm for Pt-Fe/GNPs and 2.1 nm for Pt-Cu/GNPs. Raman spectroscopy was used to find I_D_/I_G_, the ratio between the intensities of the D band and the G band, a measure commonly employed to characterize the defect concentration in samples of graphene. The I_D_/I_G_ values of the Pt-Ni/GNPs, Pt-Fe/GNPs and Pt-Cu/GNPs catalysts were discovered to be 0.230, 0.340 and 0.470, respectively [1].

Xue et al. used the thermally assisted method to anchor Pt nanocrystals onto three-dimensional (3D) porous boron and nitrogen double-doped reduced graphene oxide–carbon nanotube frameworks (Pt/BNrGO-CNT) [32]. They used the solvothermal method to obtain different BNrGO/CNT feeding ratios in the 3D Pt/BNrGO-CNT catalysts. To obtain this result, a mixture of CNT powder and GO solution NH_4_[BF_4_] is reacted for 24 h at 180 °C inside a high-pressure reactor. Afterward, a formulated (BNrGO)_5_-(CNT)_5_ support material is inserted into the ethylene–glycol mixture containing K_2_PtCl_4_, and additional processing at 120 °C is performed for a duration of 12 h in order to obtain the final product. Another ration of BNrGO-CNT was also prepared. The results obtained through ICP-MS determined the Pt loadings for the Pt/(BNrGO)_3_-(CNT)_7_, Pt/(BNrGO)_5_-(CNT)_5_, Pt/(BNrGO)_7_-(CNT)_3_, Pt/(BNrGO)_9_-(CNT)_1_, Pt/rGO, Pt/CNT and Pt/C catalysts at 18.20, 18.80, 18.70, 19.40, 18.40, 19.00 and 19.60 wt%, respectively. Pt particles show interplanar spacings of 0.223 nm and 0.195 nm, accurately corresponding to the (111) and (200) planes of face-centered cubic Pt crystals. Through TEM it can be observed that the Pt/BNrGO-CNT hybrid is composed mainly of the four types of elements (C, B, N and Pt), with these four components being uniformly distributed throughout the whole sheets. XRD reveals that the aforementioned diffraction peak appears at approximately 2θ = 25.0° for Pt/rGO and Pt/BNrGO-CNT. The three characteristic diffraction peaks of metallic Pt correspond to the (111), (200) and (220) crystal planes within the cubic Pt structure. The intensity ration between D and G (I_D_/I_G_) of the GO, rGO and Pt/rGO samples was determined as 0.87, 1.04 and 0.96, respectively, proving the presence of several topological flaws in the carbon structures. Conversely, the I_D_/I_G_ of Pt/BNrGO-CNT proved a low 0.76, hinting towards the fact that the incorporation of low-defect CNTs into the rGO skeletons can be used as a means of reducing the density of defects of the hybrid system. The specific surface areas are 226 m^2^ g^−1^ and 240 m^2^ g^−1^ for Pt/BNrGO-CNT and BNrGO-CNT hybrids, respectively.

Grad et al. prepared Pd/rGO and Pd-Au/rGO catalysts through wet impregnation of GO using an aqueous mixture of metal salts. The resulting mixture of GO and metal ions was then processed through thermal treatment in (H_2_ + Ar) mixture [33]. The Pd/rGO catalyst was prepared using GO and PdCl_2_ solution in diluted HCl by wet impregnation, resulting in 10 wt.% palladium on rGO. The target Pd-Au/rGO with metal concentrations of 7.5 wt.% Pd and 2.5 wt.% Au was obtained similarly through wet impregnation, employing a mixture comprising PdCl_2_ and HAuCl_4_ aqueous solutions. In both cases, the impregnated samples were dried at ambient temperature followed by thermal reduction in Ar. Afterward, it was thermal treated in a (H_2_ + Ar) mixture (10 vol.% H_2_) for 30 min at 250 °C. The resulting surface areas determined using the BET method are 210 m^2^/g for Pd/rGO and 206 m^2^/g for Pd-Au/rGO. The XRD profiles for both of the catalysts display the (002) graphene reflexion situated at 23° for Pd/rGO and at 23.7° for the Pd-Au bimetallic catalyst, as well as the metal reflexions: Pd (111) at 39.9°, Pd (200) at 46.4° for Pd/rGO and one metal reflexion situated at 38.9° for Pd-Au/rGO. Size of the metal crystallite calculated by XRD is 5 nm for Pd/rGO and 3.5 nm for Pd-Au/rGO. The bimetallic catalyst displays a distance between the carbon layers of 0.378 nm, whereas the Pd/rGO catalyst displays a distance of 0.386 nm, with the medium number of graphene layers for Pd/rGO being 4 and for Pd-Au/rGO being 5.5. The burning temperature for the palladium containing catalyst is 517 °C, while the Pd-Au bimetallic has a much lower value at 447 °C. The I_D_/I_G_ intensity ratio of the D and G bands is 0.90 for Pd/rGO and 0.85 for Pd-Au/rGO; values were obtained from Raman spectra [33].

### 2.3. Microwave Irradiation Method

**Figure 4:**
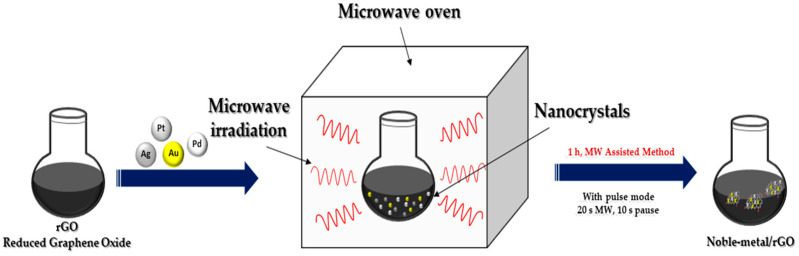
Microwave irradiation synthesis of noble metal nanocomposites.

In recent years, microwave irradiation has been used as an eco-friendly method in the synthesizing organic, inorganic and inorganic–organic hybrid materials due to its well-known advantages over conventional synthetic methods. The size as well as distribution of NPs synthesized using the light or microwave irradiation method could be easily controlled compared to reductant-assisted or thermal-assisted reduction method, by changing the intensity, power and irradiation time of the light or microwave (Figure 4). Another important property of microwave irradiation synthesis is that along with the reduction in metals, simultaneous reduction in graphene oxide is possible [13].

Wojnicki et al. synthesized Au/rGO. They first dissolved metallic gold in aqua regia to obtain the Au(III) chloride complex [34]. A Magnum II (Ertec, Poland) 600 W microwave-heated digestion system was used to obtain Au/rGO. The parameters of the microwave-heated digestion system were set to a temperature of 523 K using microwaves at a frequency of 2.45 GHz for 10 min. The pressure in the reaction vessel increased from atmospheric to approximately 40 bar. XRD showed that the intensity of diffraction lines ascribed to GO (001) and graphite (002) crystal planes was much smaller when compared to the intensity of Au (111) line. The average value of the AuNPs diameter was calculated at 12 nm. The high-resolution Au spectrum confirmed the presence of metallic gold by XPS. XPS peaks were ascribed to Au 4 f_7/2_ with a binding energy 84 eV and spin-orbit energy shift of 3.7 eV. The ratio of the intensities of the D and G bands (I_D_/I_G_) presented by the Raman spectra of the investigated samples proved to be equal to 0.60 for GO, 0.63 for rGO and 0.81 for Au/rGO, respectively. The obtained I_D_/I_G_ values are directly proportional with the number of structural defects in the sample.

Gold nanoparticles decorated on rGO were prepared using the microwave-assisted process (MW). The procedure has demonstrated remarkable advantages as eco-friendly method for Au/rGOs obtaining with simultaneous reduction in graphene oxide and formation of gold nanoparticles by an innovative one-step process. The characterization of prepared samples demonstrated good chemical stability and controllable morphology. The samples were used for membrane electrode assembly development and tested in operation of proton exchange membrane fuel cells. The electrochemical stability of the innovative Au/rGO-based cathode was analyzed using several accelerated stress tests (ASTs) by considering the cycling potential protocol. The electrochemical analysis considering the I-V study, cyclic and linear voltammetry has provided improved performances in comparison with the standard commercial cathode. The aggressive AST indicated an excellent stability; thus, the authors reported an improved electrocatalyst for oxygen reduction reaction with higher stability and durability for fuel cells. Moreover, the paper indicates the possibility of extending the protocol using the microwave-assisted process for obtaining other noble metal nanoparticles supported on rGO [35,36,37,38,39].

A single-step route to obtain platinum/platinum-cobalt uniformly distributed nanoparticles supported on reduced graphene oxide was developed recently. This route provides significant advantages such as its low cost, low time-consuming nature and high yield in comparison to state-of-the-art chemical methods used to prepare efficient Pt/rGO catalyst. The morphology of prepared samples has been evaluated by specific techniques, while the electro catalytic durability has been evaluated using the electrochemical performances in fuel cells [40,41]. Significant performance and stability in PEM fuel cells was demonstrated. The produced Pt-rGO-based membrane electrode assemblies were studied for stability under fuel starvation in comparison with commercial Pt/C-based membrane electrode assemblies. The electro-chemical activity was studied and the electrochemical response indicated the higher stability during degradation test under fuel starvation in comparison with commercial Pt/C catalyst. These results extend the applicability of described preparation protocol to other noble/transition metal nanoparticles supported on graphene-based materials.

### 2.4. Ultrasonication Method

**Figure 5:**
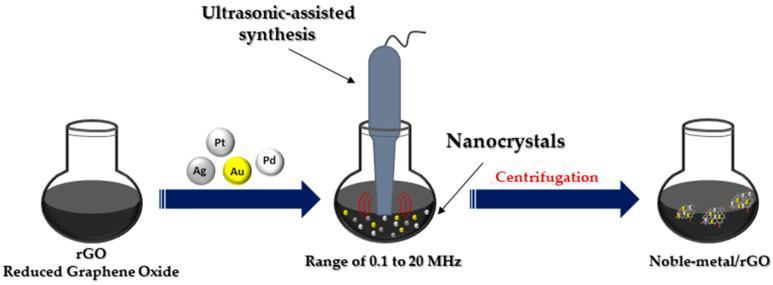
Ultrasonic-assisted synthesis of noble metal nanocomposites.

The ultrasonic method (Figure 5) leads to the rapid heating of the liquid to temperatures of 5000 K in a few nanoseconds, resulting in microbubbles with an effective effect. These microbubbles act as chemical reactors. Oxidative and reducing radicals are generated in the cavitation effect during sonolysis. Sonication in the range of 20 to 1000 kHz leads to the formation of MNPs from metal precursor solution. The collapse of these microbubbles leads to the generation of high temperatures inside the bubbles [18]. Ultrasonic testing techniques are widely accepted for testing materials in many industries, including power generation, steel, aluminum, titanium production, airframe manufacturing, jet engine manufacturing and shipbuilding [42].

Li et al. synthesized the AuPs/rGO through a mixture of 4 mg/mL GO suspension and 0.48 mg/mL AuPs solution with a volume ratio of 1:1; it was then sonicated for 1 h. After the AuPs/rGO was reduced by hydroiodic acid, it was washed with deionized water and then dried in air. The images of AuPs/rGO composite by SEM showed the uniform distribution of the AuPs on the surface of rGO. XRD measurements and the corresponding calculated results are consistent with the SEM images. The AuPs composites are inserted into the layered graphene sheets. The diffraction peak is 2θ = 8.84° for rGO to 2θ = 7.86° for AuPs-rGO. XRD measurements are consistent with the SEM images. The Raman spectra of both the AuPs-rGO and pure rGO film show a wide band at 2400 cm^−1^~3200 cm^−1^ [43].

Tran et al. prepared the Ag/GO nanocomposites through the ultrasonication method. Ag/GO nanocomposites were synthesized with GO, double-distilled water and sonicated for a duration of 10 min, after which AgNO_3_ was added. Centrifugation at 12,000 rpm was used to separate the final Ag/GO nanocomposites, which were then washed with double-distilled water [44]. The FTIR spectrum of GO reveals several proprietary peaks situated at 3224, 1724, 1226 and 1050 cm^−1^ for hydroxyl -OH, carboxyl -COOH, epoxy C-O-C and alkoxyl C-O. After anchoring with AgNPs, the epoxy stretching mode at 1226 cm^−1^ is no longer present, while peaks that are indicative of different oxygen functional groups remain well-preserved. Nanocomposites of GO and Ag/GO reveal a diffraction peak at 2θ of 10.1° in the XRD patterns relating to the (002) crystal plane of GO nanosheets. In addition to the distinctive diffraction peak of GO, the Ag/GO nanocomposites also exhibit a number of separate peaks at 2θ of 38.1, 44.2, 64.5 and 77.5 degrees, which can be attributed to the (111), (200), (220) and (311) facets of typical fcc metallic Ag (JCPDS No. 04-0783), respectively. The patterns revealed by XRD demonstrate the effective adhesion of AgNPs to GO nanowires. The I_D_/I_G_ ratio of the GO increased from 0.87 to 0.92 when the GO was anchored with AgNPs. The C/O atomic ratio found in the Ag/GO nanocomposites is greater than that of the GO, with the values obtained by XPS analysis being 2.6 for Ag/GO and 2.0 for GO. FTIR showed the characteristic peaks for Ag/GO centered at 284.8, 287.0 and 288.5 eV, respectively, for C=C, C-O and C=O. XPS analyses determined an atomic percentage of Ag of 2.46.

Bi et al. prepared PCN-222 and Ag^+^-decorated PCN-222 (zirconium-metalloporphyrinic metal–organic framework) using ZrCl_4_, meso-tetra(4-carboxyphenyl) porphine, benzoic acid and AgNO_3_. The solution was ultrasonically dispersed and dissolved in a mixture of N, N-dimethylformamide and acetic acid. Preparing the Ag^+^-decorated PCN-222@EDTA-GO-CS (CS-chitosan) foam required AgNO_3_, ZrCl_4_, H_2_TCPP and benzoic acid that were ultrasonically solvable in the N, N-dimethylformamide [45]. The Ag^+^-decorated PCN-222 showed clear crystal lattices and the TEM-EDS mappings revealed the even dispersion of Ag, C, N, O and Zr elements. The characteristic peaks of PCN-222 at 1709, 1640, 1603, 1549, 1402, 967, 801 and 719 cm^−1^ were obtained through the use of FT-IR spectra. The peaks in binding energies at 367.5 eV and 373.5 eV arose from the presence of Ag+ ions.

Mehmandoust et al. prepared Pt/CQDs@rGO nanocomposite (CQDs—carbon quantum dots) by ultrasonication method [46]. The Pt/CQDs@rGO nanocomposite is prepared using the GO dispersion, aqueous sodium citrate and ammonia solution for the CQDs, following which H_2_PtCl_6_ was added to the solution. In the XRD patterns, the characteristic peaks of (002) obtained at 11.0° (d002 = 0.85 nm), 28.9° and 24.0° were for GO, CQDs and Pt/CQDs/rGO, respectively. After the reduction process, the diffraction peaks are observed at, respectively, 46.9°, 55.7° and 81.6°, corresponding to (111), (200) and (220) planes of the face-centered cubic (fcc) structure of Pt. The crystallite sizes of GO, CQDs and Pt/CQDs@rGO are 54 nm, 17 nm and 38 nm, respectively. The large crystallite size of Pt/CQDs@rGO shows the aggregation of Pt nanoparticles. D and G bands have been detected using Raman spectroscopy at 1350 cm−1 and 1580 cm^−1^ for graphene oxide, 1350 cm^−1^ and 1580 cm^−1^ in the case of GQDs and at 1357 cm^−1^ and 1590 cm^−1^ for Pt/N-CQDs@rGO nanocomposite. The values of the ratios between the intensities of the D and G bands (I_D_/I_G_) are 0.88 and 0.94 and have been calculated for GO and Pt/CQDs@rGO, respectively. The EDX investigation exhibits all essential elements such as C, O, Na, Cl and Pt individually from Pt/CQDs@rGO nanocomposite.

Mariappan et al. presented the study of Ag/rGO prepared with glucose, vitamin C and NaBH_4_ as reducing agents through the ultrasonication method [47]. The Ag/rGO is prepared using GO, polyvinylpyrrolidone is dispersed in double-distilled water by continuous sonication for 2 h and afterward, AgNO_3_ and glucose are added. The same experimental procedure is repeated for vitamin C and NaBH_4_. The Ag/rGO samples have peaks at 2θ values of 38.13°, 44.34°, 66.44° and 77.44°, which agree with the cubic crystal structure of the Ag NPs. The grain sizes of the Ag NPs are 28 nm, 25 nm and 22 nm for GAg_G, GAg_V and GAg_S, respectively. Through Raman spectroscopy, a graphitic band (G band) at 1590 cm^−1^ and a disorder band (D band) at 1365 cm^−1^ can be observed. The absorption bands at 257 nm for GAg_G, 268 nm for GAg_S and 270 nm for GAg_V. I_D_/I_G_ ratios have been measured at 1.26 for GAg_S, 1.19 for GAg_V and 0.94 GAg_G.

Aljafari et al. prepared Pd/GO using the sonication technique. This process used GO, palladium acetate and glucose. The pH was adjusted using NaOH [48]. The diffraction peaks noticed at 40.1°, 46.6°, 68.0°, 82.1° and 86.4° match with Pd (111), Pd (200), Pd 220), Pd (311) and Pd (222), respectively. The absorption bands for Pd NPs are 260 nm and for Pd/rGO are 265 nm.

Mao et al. [49] synthesized the graphene oxide sheets decorated by silver nanoparticles using the sonication method. This process used graphene oxide colloid, AgNO_3_ and cetyl trimethylammonium bromide. The final product is graphene oxide sheets that are decorated by silver nanoparticles. The peaks at 38.31°, 44.41°, 63.51° and 77.71° can be assigned to the (111), (200), (220) and (311) crystalline planes of silver, respectively, which shows that the silver nanoparticles are composed of pure crystalline silver. The particle diameter of silver nanoparticles is about 10 nm. The band of silver nanoparticles is at about 414 nm.

For the preparation of graphene materials doped with metal nanomaterials, the synthesis methods are very important to ensure the best dispersion of the metal particles and the narrowest distribution of their size, because both significantly affect the electrocatalytic activity.

In Table 1 we presented the synthesis method according to the noble metals used. In conclusion, we can state that the chemical reduction method is specific to the noble metal Pd, the thermally assisted method is specific to the noble metals Au, Ag and Pt, the microwave-assisted method is specific to the noble metals Au and Pt and the ultrasonication method is specific to all four noble metals present in our study (Au, Ag, Pt and Pd).

## 3. Advantages and Disadvantages of the Synthesis Methods of Noble Metals Functionalized on Graphene Oxide

In recent years, different methods have been proposed for the synthesis of nanoparticles deposited on a graphene support. The choice of the most suitable method has the greatest importance in terms of the structure and catalytic efficiency of the catalysts. Table 2 presents the advantages, disadvantages and applications of the most known methods used in the synthesis of nanoparticles deposited on a graphene support.

In conclusion, the most valuable method among the preparation methods of graphene-deposited nanomaterial catalysts is microwave field irradiation, especially due to the short synthesis time, the fast and uniform heating and the significant challenge in controlling uniformity of the metal nanoparticle’s decoration on the graphene surface. By applying irradiation in the microwave field, under the influence of temperature, homogeneous reaction centers are formed in the reaction medium at the interface between the irradiation-sensitive graphene support and the metal precursor. Additionally, the presence of a reducing agent in the reaction medium means that the precursor can be converted to its metallic form by microwave irradiation.

**Figure 6:**
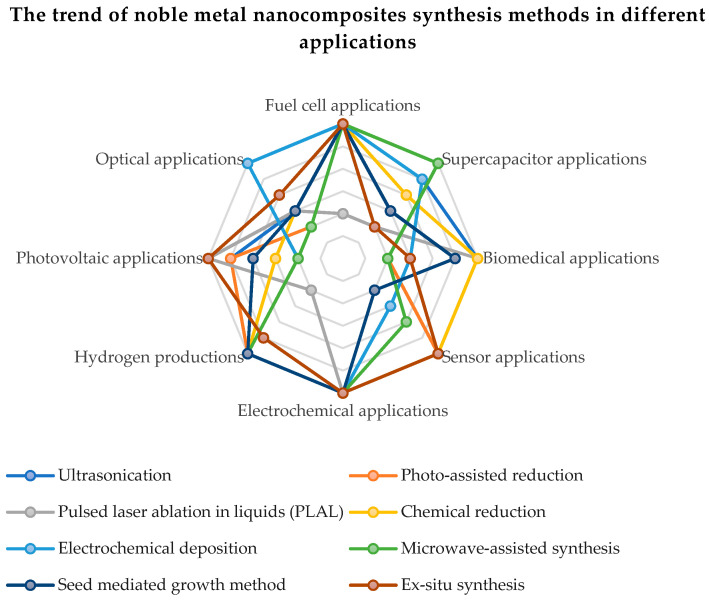
The trend of noble metal nanocomposites synthesis methods in different applications.

The qualities of noble metals have demonstrated a special efficiency in the electrocatalytic activity and the electrochemical stability of compounds based on carbon and graphene oxide. In order to improve the oxygen reduction reaction (ORR) and the quality of hydrogen adsorption and desorption, a higher electrochemical active surface area (ECSA) of the catalyst based on noble metals is necessary. The intrinsic increase in the active surface is proportional to the metal content in the chemical compound and to the dispersion of metal nanoparticles on the rGO sheets. The uniform distribution and surface morphology of noble metal nanoparticles on rGO have an effect on the ORR. An excessive reaction energy can cause an agglomeration of the noble metal nanoparticles, leading to particle sizes over 10 nm and the suppression of catalytic activity by reducing the active surface. Figure 6 present the trend of noble metal nanocomposites synthesis methods in different applications. Thus, it can be seen that the most applications of graphene functionalized with noble metals are in applications with fuel cells, renewable energy sources (photovoltaics, production of green hydrogen) and supercapacitors.

## 4. Conclusions

This review presents the most used and up-to-date methods for the synthesis of graphene functionalized with noble metals (Pt, Ag, Pd and Au) as well as the relevant methods for the characterization of catalysts. The potential of capitalizing on the improved catalytic properties of graphene functionalized with noble metals was also discussed. The studies presented in this review were carried out in order to understand how the metal–support interaction drives chemical catalysis. The preparation technique, the type and amount of metal, the nature of the support, the type of dopant and the technique of applying the catalyst, all these are dependent on the metal–support relationship. Following this review, it was found that the noble metals demonstrated a special efficiency in the electrocatalytic activity and the electrochemical stability of the compounds based on carbon and graphene oxide. It was also observed that to improve the oxygen reduction reaction (ORR) and the quality of hydrogen adsorption and desorption, a higher electrochemical active surface area (ECSA) of the noble metal catalyst is required. The uniform distribution and surface morphology of noble metal nanoparticles on rGO were found to have an effect on the ORR. Most applications of noble metal functionalized graphene are in fuel cells, renewable energy (photovoltaic, green hydrogen production) and supercapacitor applications.

## Figures and Tables

**Table 1:** Synthesis method depending on the nature of the introduced metal.

Metals	Method	References
Pt_40_Ni_60_/rGO	Chemical reduction	[24]
(Co_6_)Ag_0.1_Pt_0.9_/rGO		[25]
PdNPs		[26]
PdIrO/NGs		[27]
Pd-rGO		[28]
Au@PdNPs		[29]
AgZnO-rGO	Thermally assisted method	[30]
AuMn_3_O_4_-rGO		[31]
Ag/CeO_2_-rGO		[15]
Pt-Cu/GNPs		[1]
Pt-Ni/GNPs		[1]
Pt-Fe/GNPs		[1]
Pt-BNrGO		[32]
Pd/rGO		[33]
Pt-Au/rGO		[33]
Au/rGO	Microwave-assisted method	[34,35,36,37,38,39]
Pt/rGO		[40,41]
Au/rGO	Ultrasonication	[43]
Ag/GO		[44]
Ag-PCN-222		[45]
Pt-CQDs/rGO		[46]
Ag/rGO		[47]
Pd/rGO		[48]
Ag/rGO		[49]

**Table 2:** Advantages and disadvantages of the synthesis methods of noble metals functionalized on graphene oxide.

Method	Advantages	Disadvantages	Application	Ref.
**Ultrasonication**	▪ High temperature (5000 K) in a few nanoseconds; ▪ Friendly environmental conditions; ▪ Dual frequency conditions are used to substantially reduce the likelihood of any physical damage to the graphene sheets.	o Low concentration of GO suspension for overcoming the activation energy barrier; o Particle size dimensions are difficult to control during ultrasonication.	- Fuel cell application; - Biomedical application; - Electrochemical sensor applications.	[50,51,52,53]
**Thermally assited method**	▪ Possibility of variation of the electronic properties of the graphene oxide photoreduction composite; ▪ Photo-electrochemical reduction process is simple and also inexpensive; ▪ Increase in charge redox reaction and ion diffusion via photovoltaic effect.	o The level of reduction in certain groups on the surface of graphene oxide is not very relevant.	- Supercapacitor application; - Hydrogen production; - Electrochemical sensor applications.	[54,55,56,57]
**Pulsed laser ablation in liquids (PLAL)**	▪ Could provide a green synthesis strategy of GO metal nanocomposites; ▪ Short reaction time—from several hours to a few minutes; ▪ Does not involve toxic chemicals.	o Lack of optimized parameters of the PLAL method for improving the yield and desired properties of carbon nanomaterials; o The importance of the effect of the liquid carrier medium on the GO optical properties.	- Biomedical application; - Photovoltaic applications; - Electrochemical sensor applications.	[58,59,60,61]
**Chemical reduction**	▪ Uses green reductants; ▪ Low cost implementation.	o High loss of mechanical integrity of freeze-dried nanosheets; o Presence of a quantity of metal impurities after reduction.	- Biomedical application; - Fuel cell applications; - Sensor applications.	[62,63,64,65]
**Electrochemical deposition**	▪ Simple, efficient and fast technique; ▪ Can control the size of noble metal nanoparticles and the deposition time.	o Moderate–intrinsic electrocatalytic properties.	- Optical application; - Fuel cell applications; - Electrochemistry applications.	[66,67,68,69]
**Microwave-assisted synthesis**	▪ Uniform and fast heating technique; ▪ Uniform dispersion of a smallest particle size; ▪ High electrocatalytic activity of nanoparticles.	o Long and time-consuming to complete a reaction; o Process set up is difficult to realize.	- Fuel cell applications; - Supercapacitors application; - Electrochemistry applications.	[70,71,72,73]
**Seed mediated growth method**	▪ Small-size nanoparticle synthesis; ▪ Well-controllable growth rate.	o Possible agglomeration and unreliable electrostatic attraction of metal precursors and GO.	- Electrochemistry applications; - Fuel cell applications.	[74,75,76,77]
**Ex situ synthesis**	▪ Present the advantages of easy filtration, good shape and size control of the nanosheets.	o Linking agent is required, because the metal nanoparticle and graphene sheets are synthesized separately; o Aggregation of the metal nanoparticles before their attachment onto the GO can be a problem.	- Electron emission applications; - Electrochemistry applications; - Fuel cell applications.	[78,79,80,81]

## Data Availability

Not applicable.
